# Progressive impairment of directional and spatially precise trajectories by TgF344-Alzheimer’s disease rats in the Morris Water Task

**DOI:** 10.1038/s41598-018-34368-w

**Published:** 2018-11-01

**Authors:** Laura E. Berkowitz, Ryan E. Harvey, Emma Drake, Shannon M. Thompson, Benjamin J. Clark

**Affiliations:** 1 1 University of New Mexico, Department of Psychology, University of New Mexico, Albuquerque, NM 87131 United States; 2 90 North Patterson Avenue, Department of Psychology, Miami University, Oxford, OH 45056 United States

## Abstract

Spatial navigation is impaired in early stages of Alzheimer’s disease, and may be a defining behavioral marker of preclinical AD. A new rat model (TgF344-AD) of AD overcomes many limitations of other rodent models, though spatial navigation has not been comprehensively assessed. Using the hidden and cued platform variants of the Morris water task, a longitudinal assessment of spatial navigation was conducted on TgF344-AD (n = 16) and Fischer 344 (n = 12) male and female rats at three age ranges: 4 to 5 months, 7 to 8, and 10 to 11 months of age. TgF344-AD rats exhibited largely intact navigation at 4–5 months, with deficits in the hidden platform task emerging at 7–8 months and becoming significantly pronounced at 10–11 months of age. In general, TgF344-AD rats displayed less accurate swim trajectories to the platform and searched a wider area around the platform region compared to wildtype rats. Impaired navigation occurred in the absence of deficits in acquiring the procedural task demands or navigation to the cued platform location. Together, the results indicate that TgF344-AD rats exhibit comparable navigational deficits to those found in individuals with preclinical-AD.

## Introduction

Alzheimer’s disease (AD) is the most common form of dementia in the United States and is characterized by progressive cognitive decline and neurodegeneration^1^. Regions succumbing to AD pathology are critical to spatial navigation^2^ and correlate to the subtle navigation impairments found in preclinical and early-AD. Indeed, mounting evidence suggests that spatial disorientation, sometimes referred to as wandering, are among the earliest memory complaints in AD^3–8^. In general, disorientation is characterized as deficient localization of hidden goals^9^, a loss of direction sense^10–13^, or an impairment in correctly identifying familiar spatial scenes after a small change in view-point^4,5^. Deficits in establishing or utilizing map-like (allocentric) frameworks for navigation are frequently linked with the earliest stages of AD, while later stages are associated with deficits in simpler forms of navigation, such as approaching cues, or utilizing egocentric movements to guide behavior^9^. Navigation can therefore serve as an early marker of AD, with some studies indicating that disoriented patients are more likely to convert from the amnesic subtype of Mild Cognitive Impairment to an AD diagnosis^4,14^.

A recent animal model of AD, called the TgF344-AD rat, progressively develops a comprehensive profile of AD pathology and shows promise for elucidating the neurobiological basis of spatial disorientation in AD. Importantly, TgF344-AD rats were developed by injecting rat pronuclei with the “Swedish” mutant human amyloid precursor protein (APPsw) and Δexon9 mutant human presenilin-1 (PS1 ΔE9)^15^, and display characteristic plaque formation, endogenous tangle pathogenesis, as well as neuroinflammation, neurovascular dysfunction and cell loss^15,16^. Although the spread of pathology in this model requires further analyses, such as the evaluation of thalamic and brain stem regions also implicated in AD^2^, current studies report a similar pathological profile of that found in humans^15,17^. For example, elevated amyloid, tau, and activated microglia pathology can be detected within both the entorhinal cortex and hippocampus at 6 months of age, and by 16 months of age, the expression of amyloid and tau increase substantially. Additionally, a recent study has reported that reductions in the strength of excitatory transmission can be detected at entorhinal-to-dentate gyrus synapses as early as 6 months^18^. These synaptic changes precede reductions in neurotransmission at CA3-CA1 hippocampal synapses, which begin around 9 months of age. This emerging pattern of pathogenesis and altered signaling along the Trisynaptic circuit is consistent with the human condition^19,20^.

Both human and rodent studies have shown that pathological markers of AD predict changes to both behavioral and cellular correlates of spatial navigation. Rodents studies have extensively characterized the properties of cells that correspond to spatial features^21^, including grid cells in the medial entorhinal cortex and place cells in the hippocampus. A recent study in mice found that tau-pathology in the medial entorhinal cortex has been shown to result in dysfunction of grid cells as well as spatial memory deficits^22^. In addition, plaque pathology has shown to correspond to decreased spatial information and stability of place cells recorded in the hippocampus^23,24^. Mably and colleagues^24^ also found altered rhythmic coordination of hippocampal place cells in the 3xTg mouse model of AD, whereby place cells exhibited decreased phase locking with theta or slow gamma cycles. Currently, only two studies have assessed spatial firing or local field potential (LFP) activity in the TgF344-AD rat model^25,26^. Galloway and colleagues found that CA2/CA3 hippocampal place cells recorded in 12–20 month old female TgF344-AD rats exhibited decreased spatial fidelity relative to WT rats, though place cells from the CA1 region exhibited firing patterns similar to that of controls. Furthermore, these rats exhibited spatial deficits in an object-place task with spared object recognition indicating that spatial systems may be selectively impaired. Additionally, Stoiljkovic and colleagues^26^ found that theta power computed from hippocampal LFP, which is implicated in spatial memory performance^21^, is reduced in both male and female TgF344-AD rats^26^. Importantly, both entorhinal and hippocampal regions develop pathology early on in humans with AD^19,20^ and functional evaluation of individuals with aMCI or AD links dysfunction of these regions with spatial navigation impairments^27^. A recent study by Kunz and colleagues^28^ found that participants at risk for developing AD demonstrated reduced grid-like representations in the entorhinal cortex and elevated task-related activity in the posterior hippocampus, both of which were predictive of spatial memory performance and navigation behaviors during a virtual navigation task. Thus, overlap in pathological spread between TgF344-AD rats and humans as well as the altered signaling of grid and place cells resultant form AD-like pathology begs the question as to how TgF344-AD rats behave during spatial tasks.

Currently, only a small number of studies have investigated the time-course of spatial memory impairment in the TgF344-AD rat model^15,17,29^. Cohen and colleagues^15^ were the first to investigate spatial reference memory and spatial reversal learning using the Barnes maze in TgF344-AD rats. This study found that the amount of reference memory and reversal learning errors significantly increased at 15 months with modest increases at 24 months of age. However, Vorhees and colleagues^30^ reported that TgF344-AD rats’ latency to reach the correct escape hole during Barnes maze was like that of controls at 16 months of age, with reversal learning impairments in the Morris Water maze appearing at 24 months of age. Spatial memory in earlier age cohorts appear to be similarly less clear. Some studies have reported that TgF344-AD rats are largely unimpaired in navigating to a fixed, hidden goal location between 4 and 6 months of age^15,29^, while others have reported deficits at 6 months of age^17^. The variability between studies could be related to subtle differences in performance by male and female TgF344-AD rats, which are often pooled into a single group. Sex differences, in spatial behavior in AD, have been reported in other rodent models of AD^31–33^, and is additionally supported by the fact that females exhibit a disproportionally higher rate of conversion to AD and increased severity of clinical dementia. Furthermore, given the cross-sectional design of previous studies, and the fact that TgF344-AD display differing locomotor behaviors as early as 6 months of age^15^, non-spatial variables associated with procedural learning could also play a role. Finally, only gross measures of behavior, including the number of reference memory errors or latency to find a hidden goal location, have been used to assess spatial memory in TgF344-AD rats. Although these measures are helpful in forming a global picture of spatial memory performance, they fail to provide a detailed picture of how an animal with burgeoning pathology solves a spatial challenge.

**Figure 1:**
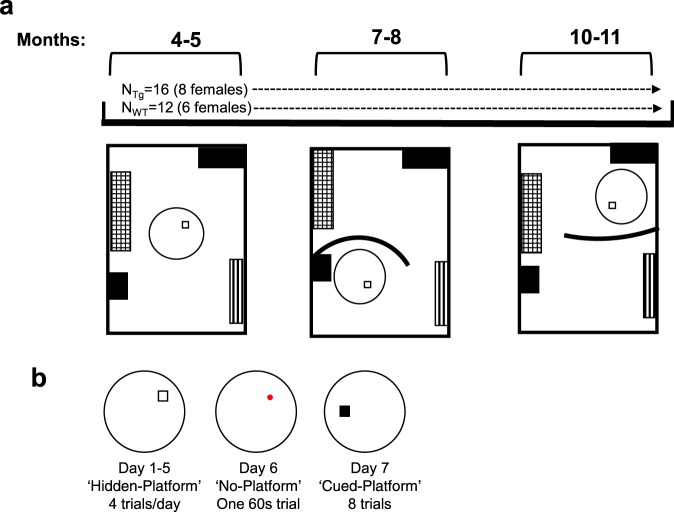
Study Time line. (**a**) TgF344-AD (n = 16) and wild-type F344 rats (n = 12) underwent testing at three time-points (shown in months). Room and maze layouts are placed column-wise below each time-point. Furniture is represented by patterned and solid squares. The black arch represents a black curtain that was used to alter room dimensions in the 7–8 and 10–11 month time-points. (**b**) The temporal order of testing consisted of 5 days of training followed by a no-platform probe trial and finally a cued-platform test on day 7. White square = hidden platform, red circle = platform area, black square = cued-platform. Pool and platform locations changed between age points.

The overall aim of the present study was to characterize spatial navigation in TgF344-AD rats at early stages of disease development. Individuals with AD may use compensatory behaviors to account for burgeoning spatial disorientation during preclinical stages^34^. To mimic this important factor, a longitudinal design was employed to assess the animals’ swim movements given prior experience and developing pathology. In addition, longitudinal designs control for age-effects commonly attributed with procedural task demands in water maze tasks^35,36^, revealing deficits that are dissociable from those expected with normal aging. Both male and female subjects were tested at three age ranges starting from 4 to 5 months, 7 to 8 months, and 10 to 11 months in the hidden and cued platform variants of the Morris water task (Fig. 1)^29,37–39^. To assess the animal’s navigation, swim movements were tracked within and between trials. A convolution analysis was employed to determine how switching to direct swims or becoming more efficient at less direct swims contributes to task performance. Several standard measures were also used to describe the memory performance and spatial distribution of movements in the pool, including escape latency, path length, path linearity and platform proximity. Consistent with what has been reported in human AD, we hypothesized that rats would express age-dependent impairments in the accuracy of their swim trajectories in the hidden platform variant of the Morris water task. We additionally hypothesized that these impairments would be expressed independent of motor and procedural deficits and co-occur with intact acquisition of cued platform learning.

## Results

One subject was excluded from the analyses at 10-11 months of age after developing glaucoma. Furthermore, one subject died during testing at 10-11 months of age and thus was not included in the analysis below.

### Hidden platform task

#### Swim Latency

**Figure 2:**
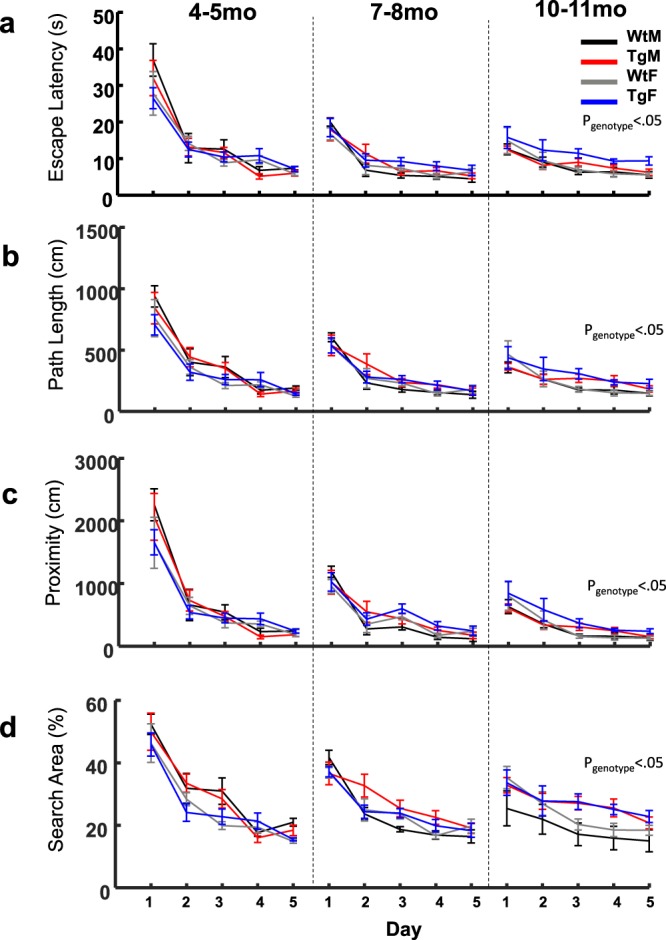
TgF344-AD rats are impaired in hidden-platform training at 10–11 months of age. (**a**–**d**) Mean escape Latency in seconds, path length in centimeters, platform proximity in centimeters, and search area in percentage of the total pool area explored is plotted over days at all age points. Main effect of genotype observed at 10–11 months for all measures, (p < 0.05, Mixed ANOVA). Groups distinguished by color: black = Wild Type males (WtM), red = Transgenic males (TgM), gray = Wild Type females (WtF), blue = Transgenic females (TgF). Data from Figure 1a for the the 4–5 months section has been previously published Pentkowski *et al*., 2017. Data are presented as mean +/− sem.

Figure 2a displays measures of escape latency for groups at each time-point. On average, animals in all groups showed a progressive decrease in escape latency at each age of testing, indicating that subjects could learn the location of the platform. This observation is supported by a significant day effect for all groups at the three time-points (*Fs* ≥ 9.00, *ps* ≤ 0.001). While there were no significant genotype or sex differences at 4-5 and 7–8 months of age, swim duration by Tg rats at 10-11 months of age was appreciably greater than WT animals (9.22 ± 1.05 sec and 7.49 ± 1.06 sec, respectively). This was confirmed by a significant genotype difference in escape latency at the final time-point (F(1, 23) = 5.76, *p* = 0.025). In addition, at 10–11 months of age, there was a trend in which Tg females had greater escape latencies relative to all other groups (10.54 ± 1.08 sec vs. ≤8.07 ± 1.08 sec, respectively; F(1, 23) = 3.10, p = 0.092).

#### Path length

Measures of path length mirrored that of escape latency in showing that animals reduced the length of their swims during training at each time-point (Fig. 2b). Again, a mixed ANOVA indicated significant day effects for all groups at the three time-points (*Fs* ≥ 15.01, *ps* ≤ 0.001). Sex differences in swim length were detected at 4-5 months of age in which females exhibited 64.1 ± 29.1 cm shorter paths on average (*F*(1, 24) = 4.85, *p* = 0.037), but not at 7–8 or 10–11 months of age. Although there were no genotype differences at 4-5 and 7-8 months of age, Tg rats had significantly longer paths, 253.51 ± 1.06 cm, compared to Wt rats, 201.37 ± 1.08 cm by 10–11 months (*F* (1, 23) = 5.18, *p* = 0.032).

#### Path linearity

The extent to which a path deviated from a direct swim towards the platform consistently decreased across training days for all three time-points (*Fs* ≥ 8.13, *ps* ≤ 0.001). Consistent with that of path length, females demonstrated less extreme deviations (4.26 ± 1.04) compared to males (5.33 ± 1.04) at 4–5 months of age, *F*(1, 24) = 8.22, *p* = 0.008, but this sex difference was not significant at 7–8 or 10–11 months of age. At 10–11 months of age, the paths of Tg rats deviated from a direct path more so than that of WT rats, 3.83 ± 1.06 and 2.79 ± 1.08, respectively (*F* (1, 23) = 8.95, *p* = 0.007). Though there was a trend for Tg rats to exhibit a higher deviation across days relative to WT rats, this genotype*day interaction was not significant (*F* (4, 20) = 2.34, *p* = 0.090). Overall, measurements of path linearity demonstrate that the paths of Tg rats exhibit larger deviations from a direct path at 10–11 months.

#### Spatial distribution of swim paths

The greater path length and larger deviation of path linearity displayed by Tg rats at 10–11 months of age may reflect a pattern of behavior in which movements are restricted to specific non-platform locations or distributed over wide areas of the pool. To better understand the spatial distribution of swim paths during hidden platform training, we analyzed the relative proximity to the platform and the overall search area of swim paths during each training trial (Fig. 2c,d). Mixed-ANOVAs conducted on search area and platform proximity indicated a significant day effect at each age (*Fs* ≥ 30.72, *ps* ≤ 0.001). At 4–5 months, females had significantly decreased search area, and tended to swim further away from the platform (394.02 ± 45.1 cm), on day 4, relative to males (188.02 ± 45.1 cm). However, there was no evidence of a significant genotype difference at 4–5 or 7–8 months of age. Nonetheless, Tg rats displayed wider search distances relative to the platform at 10–11 months (Fig. 2c). This observation is supported by significant genotype (*F* (1, 23) = 5.75, *p* = 0.025) and genotype by day effects (*F*(4, 92) = 2.58, *p* = 0.043). Similarly, Tg rats displayed a search area that was wider compared to WT animals at 10–11 months of age (Fig. 2d). This difference was confirmed by a significant ANOVA for genotype at 10–11 months (F(1, 23) = 4.69, p = 0.041). Collectively, these findings indicate that the swim behavior of Tg rats was less confined to the platform region and distributed to wider regions of the pool.

#### Qualitative swim trajectory analysis

**Figure 3:**
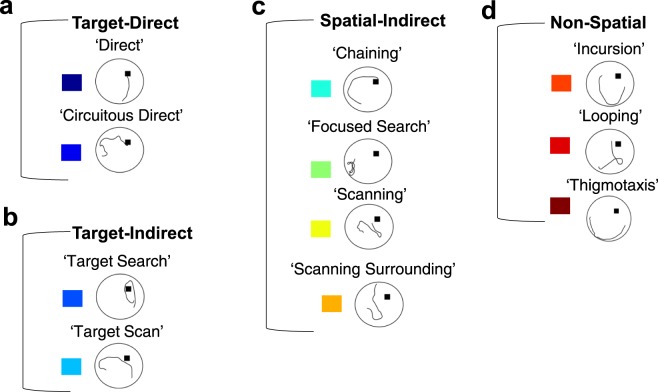
Representative examples of individual swim segments. (**a**) Target-direct (platform found within first segment). (**b**) Target-indirect (area around platform searched). (**c**) Spatial-indirect (spatial components not directed at platform). (**d**) Non-spatial (limited spatial components). See Methods for operational definitions of individual strategies.

Increases in escape latency, path length, proximity from the platform, path linearity, and search area in Tg animals at 10–11 months may be indicative of less precise swim trajectories during hidden-platform training. By evaluating how rats navigate the water maze through a qualitative segmentation analysis, we aimed to address the hypothesis that Tg animals make less direct movements towards the platform relative to WT controls, similar to behaviors identified in humans with prodromal or early-AD in real-world or virtual-world navigation tasks^14,40^. Swim paths were labeled based on 11 movement categories (Figs 3 and S1, see supplemental methods for descriptions of each category). These swim movements were then merged into 4 categories based on their directness to the platform (target-direct), a lack of directness but in proximity to the platform (target-indirect), or a tendency to organize movements in non-platform locations (spatial-indirect), or in a systematic search pattern (non-spatial).

**Figure 4:**
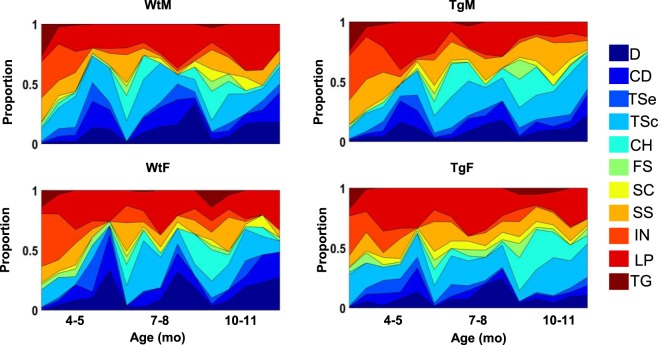
Relative proportion of swim movements over days at each age of testing. Area plots showing the proportion of all movements across days and age points. Colors indicate movement types, D = Direct, CD = Circuitous Direct, TSe = Target Search, TSc = Target Scan, CH = Chaining, FS = Focused Search, SC = Scanning, SS = Scanning Surroundings, IN = Incursion, LP = Looping, TG = Thigmotaxis. Note that Tg animals show fewer direct (D) and circuitous-direct (CD) compared to WT at 7–8 months and 10–11 months of age.

**Figure 5:**
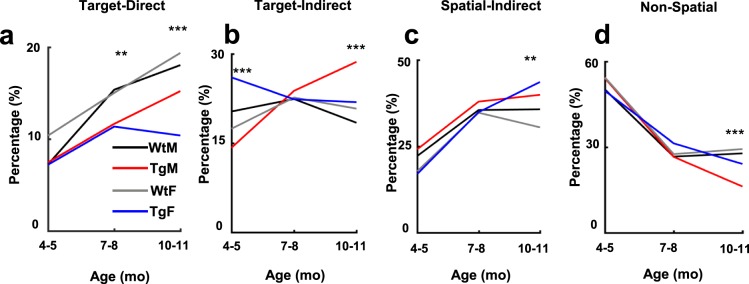
Line plots indicating the percentage of Target-Direct (**a**), Target-Indirect (**b**), Spatial-Indirect (**c**) and Non-Spatial (**d**) categories across ages. Note that Tg animals show a lower percentage of Target-Direct strategies starting at 7–8mo of age. Tg Males have a significantly higher proportion of Target-Indirect strategies at 10–11mo while Tg females use Spatial-Indirect strategies to a greater extent than WT females at 10–11mo. Tg animals show less Non-Spatial swim strategies compared to WT at 10–11 months (**p < 0.01; ***p < 0.001).

Figures 4 and 5 show the proportion of each movement category for groups across the three experimental ages and testing days. The overall proportion of each movement category at each time-point across groups is displayed in Fig. S2. As expected, both Tg and WT rats displayed an increasing number of direct trajectories as a function of training day at each age (Fig. 5a). However, the overall proportion of target-direct trajectories by Tg rats was significantly lower at 10–11 months of age (*X*^2^(1) = 12.43, *p* < 0.001). Interestingly, the proportion of direct trajectories by female Tg rats was appreciably lower compared to all other groups (*X*^2^*s* > 5.96, *ps* < 0.014). Additionally, inspection of target-direct trajectories at earlier ages suggest that Tg rats made fewer of these movements relative to WT rats. Indeed, group comparisons reached significance at 7–8 months of age (*X*^2^(1) = 5.82, p = 0.015), and although group differences were not significantly different at 4–5 months of age, Tg females made fewer target-direct trajectories compared to WT females (*X*^2^(1) = 4.07, p = 0.043). Together, these results support the conclusion that Tg rats make less directionally precise movements at 7–8 and 10–11 months of age, with a significantly lower proportion of these movements made by female Tg rats.

The reduced frequency of target-direct paths by Tg rats suggest that other movements, including those directed near the platform (target-indirect) or in other non-platform locations (spatial-indirect and non-spatial), may be favored by Tg animals (Fig. 5b–d). Consistent with this hypothesis, Tg rats were found to express a greater proportion of target-indirect movements at 10–11 months of age (*X*^2^(1) = 5.93, *p* = 0.015). Furthermore, with respect to target-indirect movements, there was a clear sex difference with Tg males showing a significantly greater proportion of these paths relative to all other groups (*X*^2^s ≥ 6.75, *ps* ≤ 0.009). Interestingly, this finding was apparent at 4–5 months, however; at that age Tg females had a greater proportion of target-indirect paths relative to all other groups (*X*^2^s ≥ 6.82, *ps* ≤ 0.009). Additionally, Tg rats performed a greater number of spatial-indirect trajectories (*X*^2^*(1)* = 14.31, *p* < 0.001) and fewer non-spatial movements at 10–11 months (*X*^2^(1) = 14.61, p < 0.001). In sum, the reduced frequency of direct trajectories by Tg rats at 10–11 months of age corresponds with an increase in spatially restricted swim paths near the platform location (target-indirect) or in other pool locations (spatial-indirect).

#### Convolution analysis

Collectively, the results described in the previous sections indicate that while general performance measures decreased across testing days, group differences were detected at 10–11 months of age. Tg rats expressed fewer direct trajectories toward the platform location at 10–11 months, and at earlier testing ages, suggesting that they utilized other movements to navigate to the platform locations. In other words, it is possible that repeated spatial training and procedural learning may have allowed Tg animals to utilize less-direct strategies that result in similar performance on standard measures. For example, an animal may become better at estimating the distance of the platform to the pool wall (i.e. chaining) over time. Thus, this movement may result in progressively shorter paths to the platform.

Given that Tg rats perform a lower proportion of directed movements (either target-direct or target-indirect movements) at 10–11 months, we tested the hypothesis that Tg rat performance is explained more so by becoming more efficient at non-direct movements rather than switching between non-direct and direct movement categories. To evaluate this hypothesis we utilized a convolution analysis^41,42^, which determines whether a change in the frequency of movement subtypes, or a change in the efficiency of swim movements, predicts trial path length (Fig. S3). First, an estimate of the frequency of each movement was calculated by dividing the number of movements in each category by the total number of path segments (Fig. S3B). Second, we estimated the efficiency of a given movement by multiplying the frequency of that movement during a trial by the trial path length. Thus, efficiency scores for a given movement subtype reflects the contribution, by that movement, to trial path length. Two models allowed us to assess the power of changes in efficiency or changes in frequency of movement subtypes in predicting trial path length. Changes in efficiency (Δ Eff) scores were derived by keeping frequency constant across trials whereas changes in frequency (Δ Freq) scores were derived by keeping efficiency constant across trials (Fig. S3C). Overall values for efficiency (i.e. average efficiency across all 20 trials) and frequency (i.e. frequency of each strategy across all 20 trials) were used as constants. Predicted path length therefore reflects the contribution of the non-constant factor on the trial path length. Individual linear regressions were then used to test each model (Fig. S3D).

**Figure 6:**
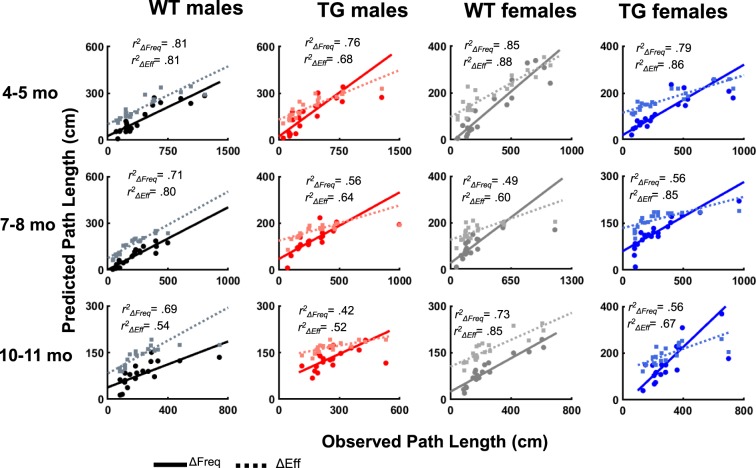
Scatter plots of derived predicted path length (cm) and observed path length (cm). Changing between movements (solid line) and their efficiency (dashed line) was significantly predictive of performance for all animals at each age of testing.

Figure 6 summarizes the regression results which indicate that switching between movement categories and becoming efficient at swim movements is significantly predictive of trial path length for all animals at all time-points. However, at 10–11 months, switching between movements (Δ Freq) takes up 17% less of the variance for Tg males and 18% less of the variance for Tg females relative to WT males and WT females, respectively (Fig. S4). Interestingly, Tg males and females exhibited differences in the predictive power of each factor. Specifically, switching between movements is a stronger predictor of performance for Tg males (*β* = 4.12, *t*(18) = 4.406, *p* < 0.001) relative to Tg females (*β* = 1.77, *t*(18) = 4.36, *p* < 0.001). Furthermore, although changes in efficiency take up a larger proportion of the variance for task performance in Tg females, relative to Tg males (67% versus 45%, respectively), changes in efficiency hold less predictive weight for Tg females (*β* = 0.993, *t*(18) = 5.72, p < 0.001) relative to Tg males (*β* = 1.80, *t*(18) = 3.67, *p* < 0.001). Overall, these results indicate that the performance of Tg animals is less associated with switching between movement categories than that identified in WT animals, supporting our hypothesis that Tg animals are more likely to improve at less efficient movements than switch to more direct movements.

#### Swim speed and non-spatial errors

To determine whether the impairments described above might be influenced by deficits in acquiring the task procedures and sensorimotor behavior, we acquired measurements of swim speed during each training trial. On average, swim speeds increased as a function of test day within each time-point (*Fs* ≥ 4.20, *ps* ≤ 0.02). In addition, female rats exhibited slower swim speeds compared to male rats - an observation that was consistent at each time-point (*Fs* ≥ 15.67, *ps* ≤ 0.001) (Fig. S5). Nevertheless, there were no significant transgenic differences as Tg and WT animals demonstrated similar swim speeds at 10–11 months of age.

We also analyzed the number of non-spatial errors per rat at each age point. The number of errors were summed across days to produce a non-spatial error score^37^. Overall, animals in both groups displayed near zero non-spatial error scores at each age of testing (Fig. S6). By 10–11 months of age, only half of the animals in each group expressed 1 or 2 errors over the 5 days of hidden platform testing, indicating an absence of procedural learning deficits in the Tg group (*X*^2^(2) = 7.54, *p* = 0.37). Thus, given the absence of clear group differences in non-spatial behaviors, it is unlikely that procedural errors contributed to the deficits described in the sections above.

#### Longitudinal evaluation of training performance

**Figure 7:**
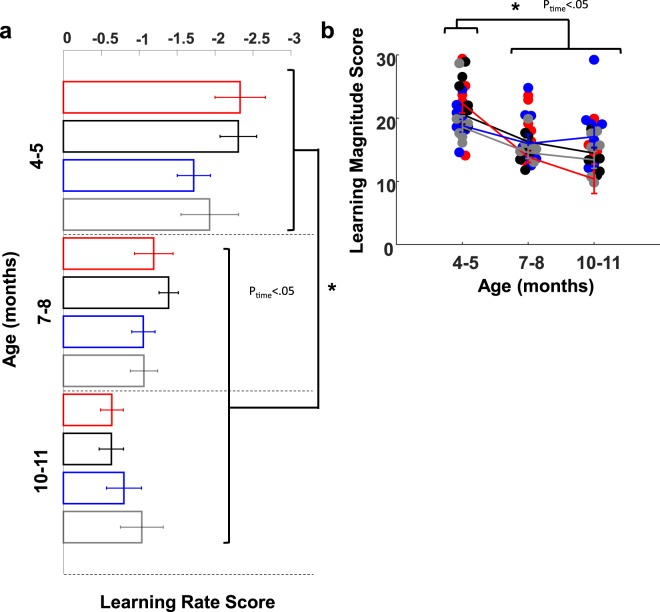
Longitudinal analysis revealed that all rats learned the platform location to a similar extent throughout aging. Learning magnitude and rate at 4–5 months was also significantly different from scores observed at latter months for all groups (p < 0.05, Mixed ANOVA). (**a**) Bar charts showing average Learning Rate Score per group show smaller changes across time. (**b**) Beeswarm plots of raw Learning Magnitude scores. Groups distinguished by color: black = Wild Type males (WtM), red = Transgenic males (TgM), gray = Wild Type females (WtF), blue = Transgenic females (TgF). Data are presented as mean +/− sem.

Spatial learning was evaluated longitudinally by assessing the rate of change and the magnitude differences of composite learning scores (Fig. 7). Composite scores were computed from the average of normalized values of swim latency, path length, cumulative proximity and search area (see Supplemental Methods). Learning rate scores that are negative indicate an improvement in task performance. While learning rates were similar between groups, (*Fs* ≤ 1.35, *ps* ≤ 0.26), all groups exhibited less negative learning rates over time, (*F*(2, 22) = 19.08, *p* < 0.001). Learning rate was steepest at 4–5 months which was −0.847 ±0.18 units steeper than the average rates at 7–8 months and −1.227 units steeper than average rates at 10–11 months (Fig. 7a). Thus, all rats exhibited progressively smaller changes in performance over time, however; all rats learned the task at the same rate. Given that all rats learn at the same rate, we next wanted to address the question whether groups exhibit similar overall task performance across time. Learning magnitude scores are computed by averaging together daily composite scores. Collapsing across days allows assessment of overall performance at a given time-point. Higher scores reflect poorer performance. Overall, all groups showed significant increases in task performance over time (*F*(2, 22) = 28.538, *p* < 0.001). Moreover, a sex by time interaction revealed that, although females showed greater performance relative to males at 4–5 months (0.187 ± 0.01 vs 0.212 ± 0.01, respectively), males outperformed females at 10–11 months (0.135 ± 0.01 vs 0.153 ± 0.01, respectively; *F*(2, 22) = 3.77, *p* = 0.039). Evaluation of learning rate and overall task performance indicate that Tg rats show similar learning rates and magnitudes on composite scores relative to WT rats during hidden platform training. Furthermore, these findings support the notion that Tg rats are successfully able to improve their task performance despite making progressively less spatially precise swim movements relative to WT rats during training.

#### No-platform probe test

**Figure 8:**
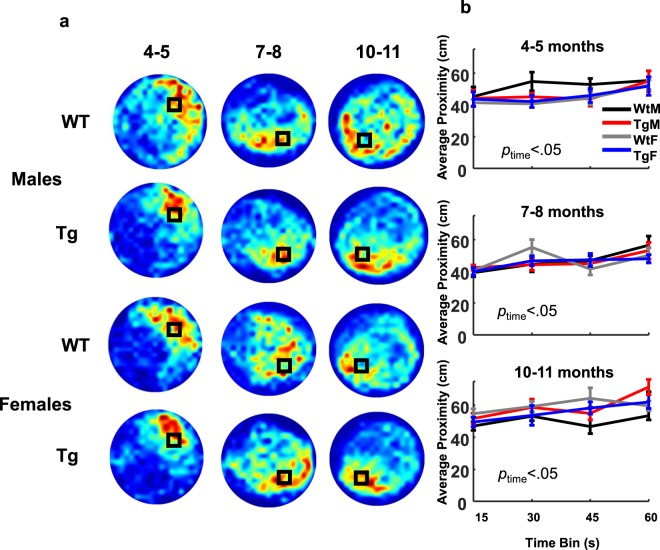
All groups exhibited similar preference for the platform location during the probe trial. (**a**) Heat maps represent weighted occupancy across the entire 60s trial. Hot colors indicate longer dwell times. The platform area is denoted with a black square. Data from the 4–5 month time-point has been previously published Pentkowski *et al*., 2017. (**b**) Average proximity away from the platform in centimeters is plotted across 15s time bins. All groups demonstrated similar average proximity within each time bin, though proximity to the platform increased as function of time (p < 0.05, Mixed ANOVA). Data are presented as mean +/− sem.

At each testing age, a no-platform probe test was conducted 24 hours after the final day 5 training trial. Figure 8a shows heat maps representing the dwell time in each location of the pool collapsed across animals in each of the Tg and WT groups. The heat maps suggest that rats from each group organized their movements around the trained platform location and spent a disproportionate amount of time near this region. To determine whether the preference for the platform region is expressed for the full duration of the probe session, we divided the analysis into four 15 second bins (Fig. 8b). At each test age, measures of average proximity increased as a function of time bin, suggesting that animals made fewer swims near the platform location by the end of each probe session (*Fs* ≥ 3.30, *ps* ≤ 0.025). However, measures of target preference score indicated significant differences at 7–8 months of age only, whereby all animals had significantly lower preference for the platform quadrant in the last 15 s versus all other time bins (*F*(3, 72) = 3.13, *p* = 0.031). Neither tests of proximity nor preference score revealed significant Tg and sex differences or interaction effects at each test age, indicating that groups displayed an equivalent search preference for the platform location by the end of training.

### Morris Water Task - Cued Platform

In the cued platform task, Tg and WT rats showed similar performance at each testing age (Fig. S7). Mixed ANOVAs conducted on escape latencies at each time-point indicated all animals had decreased escape latencies in the second trial block versus the first trial block at 4–5 months and 7–5 months (*Fs* ≥ 4.46, *ps ≤* 0.045), but only trending differences were observed at 10–11 months (*F*(1, 23) = 3.81, *p* = 0.063). Importantly, there were no group differences detected between Tg and WT groups at the three test ages. Further, there were no main effects of sex at each testing age. At 4–5 months of age, there was a significant sex by block effect, whereby female animals were slightly slower to reach the platform relative to male animals in the first trial block. Furthermore, Tg females had slightly elevated latencies to reach the platform relative to Tg males, though this genotype by sex effect only trended towards significance (*F*(1, 24) = 2.97, *p* = 0.098).

## Discussion

The primary conclusion of the present study is that clear spatial navigation impairments by TgF344-AD rats were identified at 10–11 months of age. Specifically, TgF344-AD rats spent a longer time finding the escape platform, traveled a longer distance in the pool, search a further distance away from the escape platform and searched a wider area of the pool (Fig. 2). In addition, starting at 7–8 months of age, the directionality of trajectories to the platform made by Tg rats was attenuated (Figs 4 and 5) and at 10–11 months of age switching from less direct to more direct trajectory types contributed less to task performance in Tg rats (Figs 6 and S4). Longitudinal analysis of learning magnitude and the rate of learning revealed no differences between groups, indicating that Tg rats successfully improve at the task despite using less precise swim movements (Fig. 7). While navigation impairments were detected during training in the hidden platform task, a 60 second no-platform probe test, conducted on the 6th day, indicated that both Tg and WT groups displayed a similar preference for the platform quadrant (Fig. 8). This pattern of impairment at 10–11 months supports the conclusion that although Tg animals are impaired at executing an optimal trajectory and search pattern near the hidden platform region, Tg rats can express a preference for that location by the end of the experiment.

The deficits reported in the present study were observed in the absence of group differences in sensorimotor or procedural learning. Several analyses support this conclusion: first, measures of swim speed failed to indicate significant differences between Tg and WT groups at any age of testing. This observation is also consistent with recent work^17^. Secondly, measures of non-spatial errors at each age of testing failed to indicate Tg and WT differences. Lastly, Tg and WT animals performed similarly on the cued platform task at each test age, indicating that Tg animals could learn to navigate by approaching a cue directly marking the goal. These findings strongly suggest that Tg and WT animals were equivalently capable of acquiring the non-spatial demands of the Morris water task.

The results of the present study offer some clarification regarding the time-course of spatial navigation impairment observed in this model. While the clearest navigation deficits were detected at 10–11 months of age in the present study, our results show that Tg rats displayed a significant decrease in the directness of their swim trajectories by 7–8 months of age. Notably, Tg and WT animals were equivalent in standard measures of water task performance at 7–8 months of age (i.e., escape latency and path length). One possible explanation for the latter finding is that repeated spatial training and procedural learning may have allowed Tg animals to utilize swim movements that result in similar performance on standard measures. Indeed, a convolution analysis indicated that Tg rats get better at less direct movements to find the platform than switching to more direct movements. Finally, it is important to note that previous studies report that Tg rats tend to have greater spatial difficulties in manipulations involving “reversal” tests in which the goal is moved to a novel location. Because Tg rats display intact navigation in standard tests at this age range^15,17,29^, it is possible that reversal impairments may reflect the increasing demands on behavioral flexibility rather than navigation per se. This possibility should be explored in future work.

Overall, the spatial impairment in TgF344-AD rats found here also closely correspond to those observed in individuals with preclinical or prodromal AD^6,7^. Although reference memory remained intact across all time points, Tg subjects exhibited less accurate trajectories and platform search patterns indicative of allocentric navigation impairments. In contrast, the intact cued platform navigation is suggestive of intact egocentric navigation. This pattern of impaired allocentric, and intact egocentric navigation, is consistent with impairments observed in human subjects with early AD^43^. Thus, it is likely the preclinical phase of TgF344-AD rats lies prior to 10 months, and greater spatial navigation and memory impairment would be observed later. Prior characterization of pathological markers of AD in TgF344-AD rats supports this notion^15,16,18^. The navigation differences observed at 10–11 months in TgF344-AD rats could be considered a putative MCI phase, though further characterization of pathology and behavior is needed.

Although there were no clear differences between male and female Tg rats on measures of path length, platform proximity, path linearity or search area, we did observe a trend for greater escape latency at 10–11 months of age. Additionally, our detailed path analyses indicated that female Tg rats performed significantly fewer direct trajectories toward the hidden platform at 10–11 months of age. Interestingly, performance of direct paths by female Tg rats was similar to that of male Tg rats at 7–8 months of age but not at 10–11 months of age, suggesting a potential 7-month onset of subtle changes in swim path trajectory. Male Tg rats demonstrated a slightly decreased, yet apparent impairment, in direct navigation at 10–11 months relative to WT rats. Thus, the attenuated directional deficits found in male Tg rats relative to female Tg rats may reflect sex-specific progression profiles like that found in various models of AD-like pathology^31,32,44,45^, and is consistent with human studies in which the prevalence and rate of conversion to AD is higher in females^33,46,47^. Finally, our observations suggest that detailed path analyses might have greater sensitivity at detecting group differences than general performance measures.

The hippocampus has been a strong focus in AD research, despite various other limbic circuit structural involvement in AD^2,48^. It is currently unclear whether TgF344-AD pathology emerges in other limbic regions associated with spatial navigation, such as the anterior thalamic nuclei or retrosplenial cortex. This is particularly important given cell types coding for head direction are found in both regions^49,50^, and damage to both regions can produce deficits in the directional accuracy of navigation^51,52^. Whether TgF344-AD rats exhibit AD pathology and disrupted spatial signaling in limbic-thalamic and limbic-cortical regions at early stages of development warrants investigation.

The present study found that TgF344-AD rats express clear navigation impairments at 10–11 months of age that mimic impairments found in humans with preclinical or prodromal AD. The longitudinal design provided rats with experience in a spatial task prior to the predicted onset of pathology, like that of humans exploring their environment before developing AD. This prior experience enabled a precise assessment of navigation without the inclusion of factors associated with non-spatial task demands. Additionally, the animal’s prior experience allowed for a careful dissection of how their behavior might evolve given burgeoning pathology. By using a detailed path analysis, subtle deficits in the directness of trajectories to the hidden platform were detected at earlier ages and were more sensitive to sex differences than gross behavioral measures such as escape latency, path length, search proximity, search area and path linearity. Future studies may therefore benefit in using a longitudinal approach to assessing spatial navigation in TgF344-AD rats. Finally, spatial memory was intact for all animals across all ages indicating that developing deficits observed at 10–11 months in Tg animals may be indicative of an MCI stage of disease progression. The current study was not able to determine whether the spatial navigation impairments observed in this study were predictive of AD-like pathology or vice versa. Future work would benefit from identifying whether the observed navigational impairments in this model map onto brain regions that are involved in directional computation, such as the anterior thalamus or retrosplenial cortex. Ideally, use of non-invasive methods of analysis, such as magnetic resonance imaging, should be used to preserve the ability to evaluate the same subjects over time. Overall, the TgF344-AD rat model provides substantial promise for elucidating the neurobiological mechanisms of spatial disorientation in AD.

## Methods

### Subjects

Sixteen TgF344-AD (Tg) rats counterbalanced for sex and expressing mutant human amyloid precursor protein (APPsw) and presenilin 1 (PS1ΔE9) were obtained directly from the Rat Resource and Research Center (Columbia, MO). Twelve wild type Fischer 344 (WT) rats (Harlan laboratories, Indianapolis, IN) counterbalanced for sex served as control subjects. Subjects were maintained under controlled temperature (21 ± 2 °C), were housed with Tg or WT pairs, and were kept on a 12 hour light/dark cycle (lights off at 09:00 AM). Food and water was provided ad libitum throughout the duration of the study. Animal care practices and experiments were approved by the University of New Mexico Institutional Animal Care and Use Committee and adhered to the APA ethical principles of animal use. All subjects included in this study were also used in a prior publication^29^.

### Longitudinal design

A longitudinal mixed design was used to assess changes in navigation in the hidden and cued platform variants of the Morris water tasks at three age time-points. At the start of testing, subjects’ age ranged from 4.30–5.7, 7.5–8.9, and 10.3–11.7 months at each respective time-point. For clarity, we refer to these ages throughout the remaining manuscript as, 4–5 months, 7–8 months, and 10–11 months of age. Tasks were administered in the same temporal order at each time point (Fig. 1a), whereby the hidden platform task was administered before cued platform training. Procedures were maintained between time points and experimenters did not change throughout the duration of the study. Experimenters handled the rats for as long as four weeks prior to experimentation with ~5 min of handling per day. All rats were naïve to experimentation prior to the start of the first experiments at 4 months of age. Data measures from the hidden platform task from the 4–5 month time-point, including escape latency and probe proximity/preference score, that are presented here have been previously published^29^, but has been reanalyzed to assess this measure longitudinally.

### Apparatus and testing room

A circular pool (150 diameter, 48 cm high) with a white inner wall situated on a wooden frame (50 cm high) and a 16 cm × 16 cm plastic escape platform covered with a metal grate with a height of 25 cm was used for all tasks. The pool was filled with water (20–22 °C) until the level reached 2.5 cm above the top of the platform. Non-toxic white paint (500 ml) was used to make the water opaque. Distal environmental cues were composed of various objects (movie posters, thin particle board hangings) and furniture (desks and bookshelves) and were maintained in fixed locations during training at each time-point. However, the room layout, pool, and platform location was modified at each time-point (Fig. 1b). An overhead camera was fastened above the pool to record swim behavior for subsequent analysis.

### Morris water task - hidden platform

The purpose of the hidden platform variant of the Morris water task is to assess animal navigation to a precise spatial location based on multiple allocentric spatial cues particularly those associated with the features of the distal environment^36,37,53,54^. Rats were given 4 trials per day for 5 consecutive days. Each trial consisted of randomly placing the rat into the pool facing the wall at one of four equidistant drop locations. If the rat did not locate the platform within 60 seconds, the experimenter guided the rat to the platform by hand. The rat remained on the platform for 10 seconds to allow the rat to sufficiently assess distal cues. At the end of the trial, the rat was returned to a holding cage, while the other rats in the group were tested (<9 minute inter-trial-interval). Drop location varied between time-point/trials/days, but was maintained between subjects. Subjects were run in groups of seven for each task. At the end of the 4 trials, rats were returned to their home cages in the colony room. Time to reach the platform (i.e. escape latency) was recorded by an experimenter at the end of each trial while path length, cumulative proximity, path linearity and search area were computed off-line. Measures of learning for each animal were calculated for each trial and averaged within a day.

### No-platform probe test

Twenty-four hours after the final trial of the fifth training day, a probe trial was conducted in which the platform was removed from the swimming pool. Rats were released on drop points on the opposite side of the platform quadrant. Rats were permitted to swim for a total of 60 seconds after which it was removed from the pool and placed in a holding cage.

### Morris water task - cued platform

A cued-platform test was performed to verify that animals could navigate toward a cue directly associated with the platform location^37,55,56^. In this task, a 10 cm diameter black ball with a white horizontal stripe was attached to a metal rebar and placed above the center of the platform (11.5 cm above the water). The cue was visible from any location in the pool. Subjects were trained for a single day consisting of 8 trials at least 24 hours after the no-platform probe test. Trial procedures mimicked those used for spatial acquisition in the hidden platform task described above^38^.

### Behavioral analysis

From the video records, behavioral coders blind to experimental group manually tracked each animal’s location in the pool. Tracking analysis was performed for each trial of the hidden platform and no-platform probe^29,53^ (see Supplementary Methods). From the tracking data, we were able to perform a detailed qualitative swim trajectory analysis to determine the number of directed swim movements toward the hidden platform during training^41,42,57–61^. Swim movements were coded manually by one coder based on definitions derived from the qualitative descriptions used in previous work^42,57,58,62^. Briefly, a custom Matlab script (R2017, The MathWorks, Natick, MA) automatically segmented whole paths into 200 cm increments in order to capture the use of various swim movement types within a given trial. Each segment overlapped 70 % to minimize labeling bias secondary to segment start/end points^57^. Reliability was obtained by having the coder manually label a subset of randomly selected segments that were plotted within the pool context in a matlab figure window. Each segment was plotted twice in a random order and the two labeled sets were compared using a Pearson correlation. The coder continued this process until the segments were labeled with 95 % reliability. Afterwards, the coder, blind to experimental groups, manually categorized all path segments into 11 movement subtypes (Figs 3 and S7). Segments that were indicative of multiple movement types were labeled with each respective movement. For clarity of analysis, the movements were further divided into 4 broad categories. Figure 3 provides examples of each movement subtype and analysis category (see also Supplementary Methods for detailed descriptions).

Lastly, an experimenter blind to group quantified the number of non-spatial learning errors during training trials for the hidden platform experiment. As previously described^36,63,64^, non-spatial errors included diving behavior (diving below the surface of the water during a trial), floating (the absence of swimming for more than 3 sec), platform deflections (the failure to obtain purchase onto the platform after contact), mounting errors (the failure to climb the platform after 1 sec of contacting the platform), and jumping off the platform. Each error was assigned a score of 1 and a total was obtained by summing the errors^37^.

### Statistical analysis

Statistical analyses were performed using SPSS version 26 (IBM, Armonk, NY) and Matlab. Mixed-model ANOVAs using a multivariate approach were used to assess differences within or between time-points. Between-subject factors consisted of sex (female, male) and genotype (Tg, WT). Outcome measures were evaluated in blocks of four trials (day 1, day 2, day 3, day 4, day 5) or time-point (age 4–5, age 7–8, age 10–11). Shapiro-Wilk tests were used to determine whether data were normally distributed and log transformations were applied to non-normal data. Box’s Test was used to verify equality of covariance matrices was met for all tests. Bonferroni corrections were applied when appropriate to control for multiple comparisons. Two-way ANOVAs were used to compare performance on the cued platform task within a time point using genotype and sex as between subject factors. The results of the detailed path analyses were subjected to Chi square tests. Linear regression analyses were used to determine the proportion of variance of task performance accounted for by convolved factors (described further in the Results section). Given the non-normal distribution of residuals for convolved factor regressions, a robust linear regression approach using a Tukey’s bisquare weight function was used to minimize the effect of outliers. Significance was set at *α* = 0.05 for all tests.

## Electronic supplementary material

Supplemental Information

## Data Availability

The datasets generated during and/or analyzed during the current study are available from the corresponding author on reasonable request.
