# Supplementary material for: Progressive impairment of directional and spatially precise trajectories by TgF344-Alzheimer’s disease rats in the Morris Water Task
Source: Sci Rep. 2018 Nov 1;8:16153. doi: 10.1038/s41598-018-34368-w (PMC6212523; doi:10.1038/s41598-018-34368-w)
Supplement: Supplementary file 1 — Supplemental Information [file 41598_2018_34368_MOESM1_ESM.pdf]

Progressive impairment of directional and spatially precise trajectories by TgF344-Alzheimer's disease Rats in the Morris Water Task

Laura E. Berkowitz[1], Ryan E. Harvey[1], Emma Drake[1], Shannon M. Thompson[1,2], Benjamin J. Clark[1]*

Supplementary Methods

Composite Scores. Composite scores were created to evaluate task performance over time-point based on multiple highly correlated (r>.7) task domains (i.e. time to complete the task, distance traveled, distance from platform, and percent pool area explored). Representative measures such as swim latency, path length, cumulative proximity and search area, respectively, were included in the calculations. To capture relative changes over time, individual measures were normalized from 0 to 100 across all time-points then averaged within a time-point and for each day of training. Learning magnitude was assessed by averaging across days within a given time point. Higher learning magnitude scores indicate poorer performance. To compute learning rate, composite scores at each time-point were fitted with a linear slope to determine the overall rate of change. Negative learning rate scores are representative of learning.

Video Tracking. Manual tracking was performed over automated methods to obtain precise location of the animal's head position and to reduce the incidence of tracking errors due to the poor contrast between the animal and pool water. From the video records, behavioral coders blind to experimental group manually tracked each animal's location in the pool. Tracking analysis was performed for each trial of the hidden platform and no-platform probe. First, raw video files were converted to JPEG images in a Linux bash shell using FFMPEG (https://www.ffmpeg.org). Image files were then imported into Fiji (https://imagej.nih.gov/ij/) and x- y- coordinates of the animal's nose was acquired for each video frame (10 frames/sec) using the Manual Tracking plugin. Custom Matlab (R2017, The MathWorks, Natick, MA) scripts were designed to smooth the tracked swim paths using the runline function from the Chronux toolbox (www.chronux.org). The process of smoothing has been shown to reduce the

inflation of measures dependent on path length[1].The smoothed paths were then analyzed for path length (cm), swim speed (cm/sec), platform proximity (cm), search area (%) and path linearity. Platform proximity was measured by summing the distance between the subject's location and the center of the platform location over 1 sec intervals[2]. The distance from the drop location to the center of the platform was then subtracted from the total summed distances as drop locations were variably distant to the platform. Search area was obtained by first dividing the pool surface into a matrix of 50 x 50 bins (each bin = 3cm x 3cm). Search area was expressed as the proportion of the pool visited by the animal (number of visited bins divided by the total number of bins in the matrix). Path linearity was employed to assess the magnitude in which a path meandered from a direct swim to the platform. Specifically, path linearity equaled the total path length divided by the distance from the animal's drop location to the center of the platform. Higher linearity scores indicate a larger degree of meander whereas a score closer to 1 indicates a more direct path.

For no-platform probe trials, the pool was divided into four equal quadrants and the total dwell time in each quadrant was determined. From these measures, a platform preference score was calculated by taking the average difference in dwell time between the platform quadrant and the opposite quadrant[3]. We also calculated an average platform proximity for the probe test by taking an average of the distance between the subject's location and the center of the platform location across the 60 second probe test.

Segmentation Labelling

Example movement categories are indicated in Fig. 3 & S1. Target-direct movements included trajectories that contained no looping and path lengths less than 200cm and were further defined as a direct movement to platform (i.e. direct) or meandering/arched movements towards

platform (i.e. circuitous-direct). Target-indirect movements included circling or searching next to the platform (i.e. target-search), and trajectories that passed by platform (i.e. target-scanning). Spatial-indirect movements involved patterns indicative of spatial processing but were not directed towards the platform location, such as maintaining a swim path set approximately the distance of the platform to the wall (i.e. chaining), searching of a focal area in the pool (i.e. focused-search), sustained swimming in the center of the pool (scanning), and sweeping swim paths traversing pool quadrants (i.e. scanning-surrounding). Non-Spatial movements including wall hugging (i.e. thigmotaxis), using the wall to head in a random trajectory away from the pool wall (i.e. incursion), and a path that circles over itself (i.e. looping). If a segment displayed characteristics of multiple movement types, the path was labeled with all applicable types.

Supplementary References

1. Hen, I., Sakov, A., Kafkafi, N., Golani, I. & Benjamini, Y. The dynamics of spatial behavior: how can robust smoothing techniques help? *J. Neurosci. Methods* **133,** 161–172 (2004).
2. Gallagher, M., Burwell, R. & Burchinal, M. Severity of spatial learning impairment in aging: development of a learning index for performance in the Morris water maze. *Behav. Neurosci.* **107,** 618–626 (1993).
3. Harker, K. T. & Whishaw, I. Q. Impaired spatial performance in rats with retrosplenial lesions: importance of the spatial problem and the rat strain in identifying lesion effects in a swimming pool. *J. Neurosci. Off. J. Soc. Neurosci.* **22,** 1155–1164 (2002).

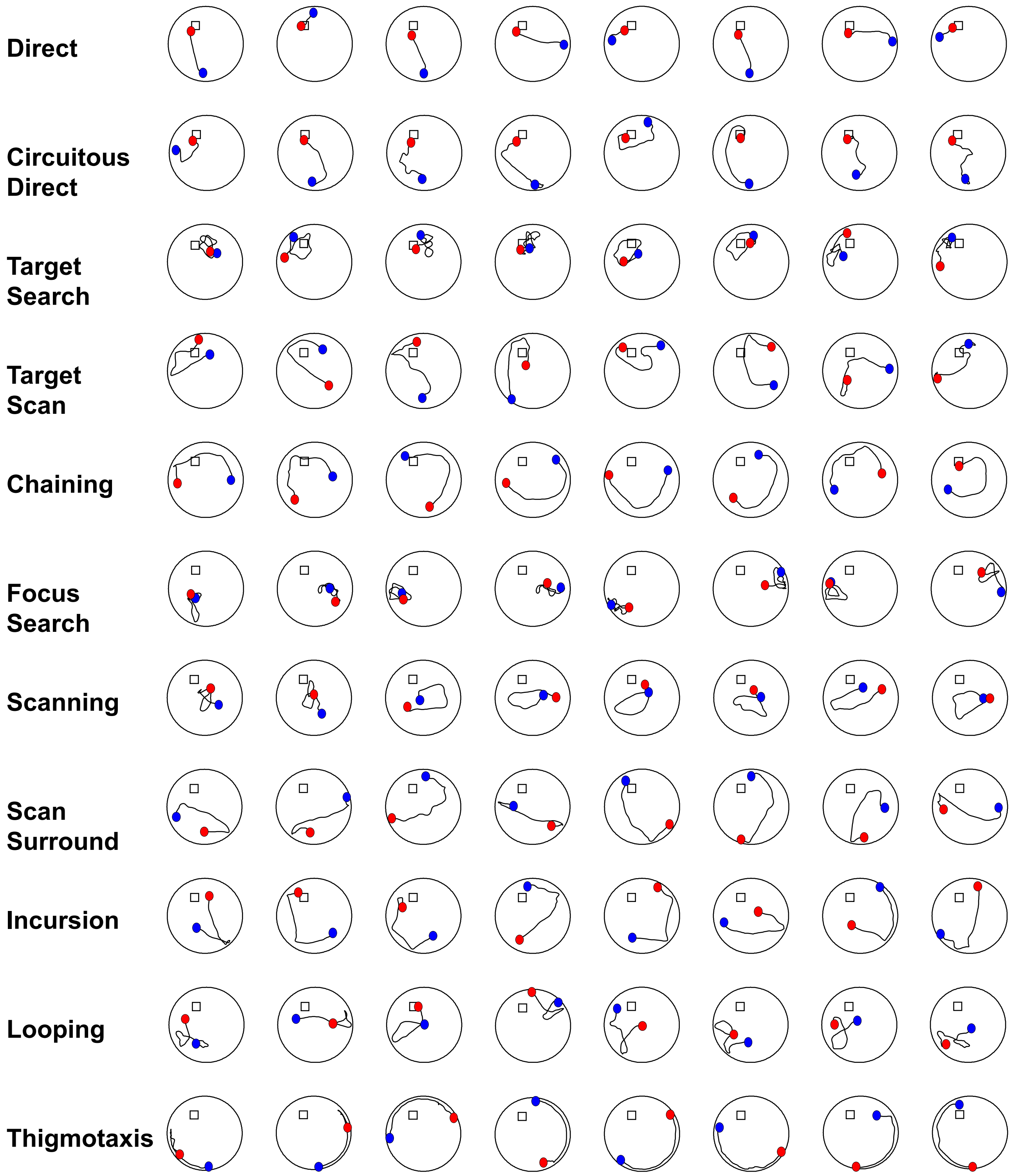

Figure S1. Example paths for each movement type. The pool edge is indicated by the black circle and platform by the black square. Start and end locations are indicated by a blue and red dot, respectively.

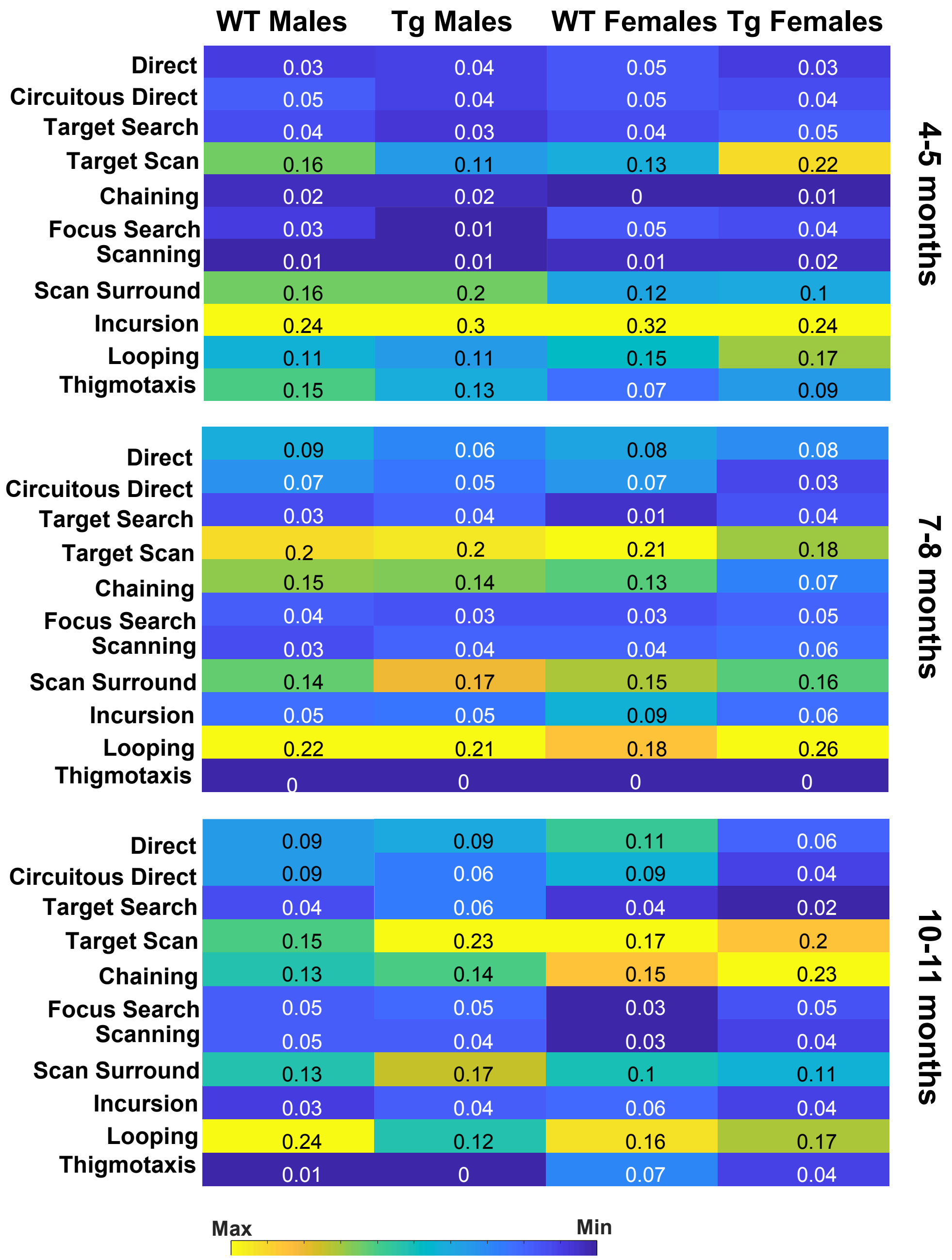

Figure S2. Heatmaps displaying the overall proportion of each movement type used during reference memory training for each time-point. Cells are normalized by the max value (yellow) for each group (column-wise) at each time-point. The trend for all animals to make more spatially contingent movements is revealed by a transition from cooler to warmer tones by 10-11 months. Note also that Tg Females show a 2% decrease in making direct paths at 10-11 months, though they increase chaining movements by 13% relative to 7-8 months. Data are presented as proportions.

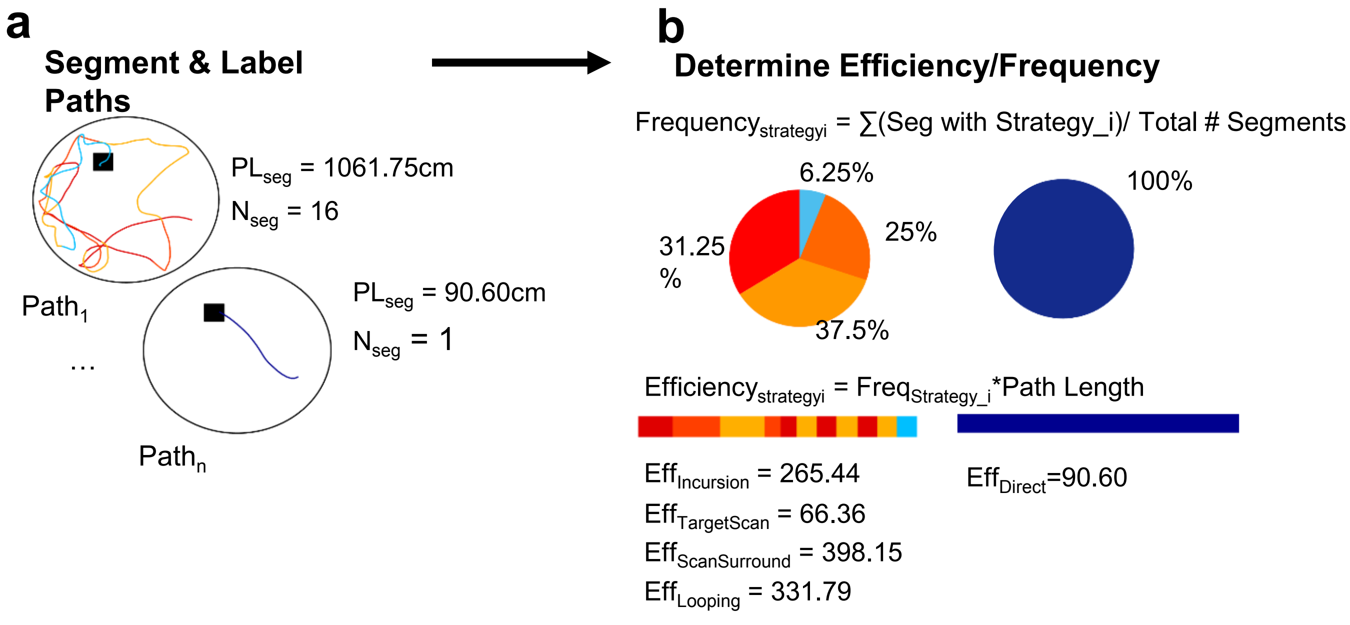

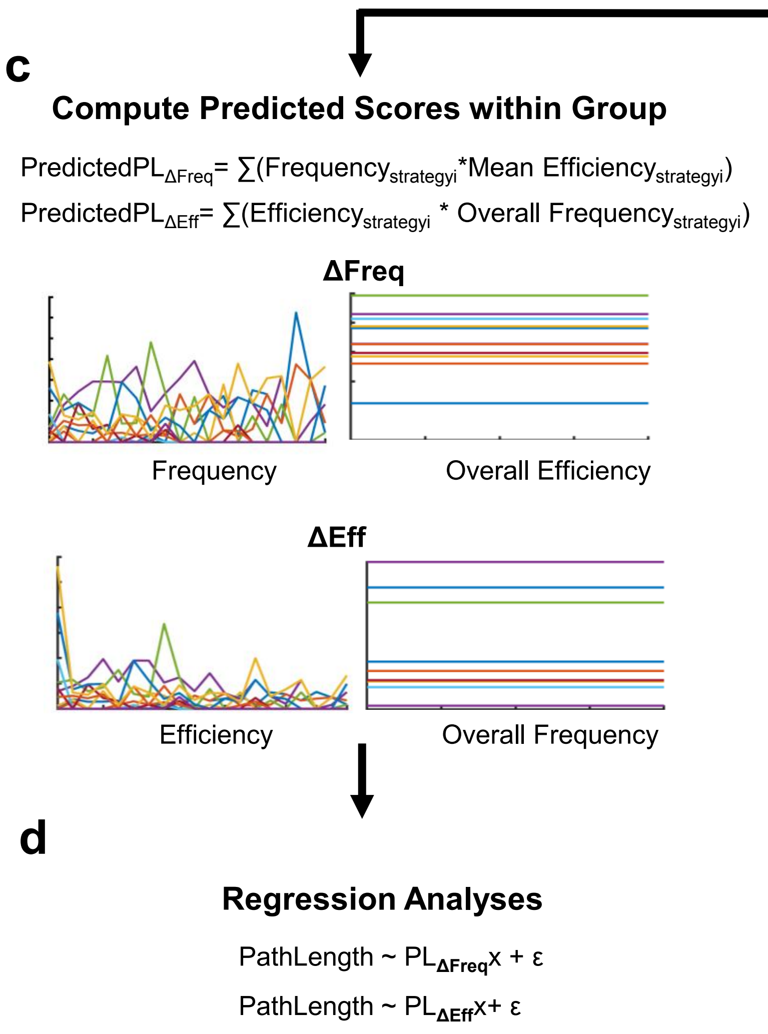

Figure S3. Analysis pipeline for assessment of swim movements using convolution analysis. (a) Example paths with movement category indicated by color, PL=path length, seg=segment. (b) Pie charts represent the proportion of a given movement within a trial. Horizontal colored bar represents the proportion of a given movement segment over time across the relative distance. Equations for computing the frequency and the efficiency of the segment and values for example paths denoted in (a). (c) Convolution analysis derived scores for frequency-fixed (Δeff represents changes in efficiency) and efficiency-fixed (Δfreq represents changes in frequency). (d) Model terms for linear regressions for each model.

		4-5mo			7-8mo			10-11mo		
Model	Group	β	p	r^2	β	p	r^2	β	p	r^2
Δeff	WT Males	2.24	***	.81	1.97	***	.80	1.86	***	.54
	TG Males	1.96	***	.68	2.24	***	.64	1.80	**	.45
	WT Females	2.99	***	.86	1.37	**	.60	2.75	***	.85
	TG Females	1.93	***	.89	3.57	***	.85	.993	***	.67
Δfreq	WT Males	3.21	***	.82	2.02	***	.71	2.32	***	.69
	TG Males	3.16	***	.77	3.77	***	.56	4.12	***	.52
	WT Females	2.95	***	.85	1.40	n.s	.49	3.01	***	.74
	TG Females	4.79	***	.79	3.66	***	.56	1.76	***	.56

Figure S4. Linear regressions indicate dynamic patterns of swim movements across testing age. Δeff= regression model of predicted score computed by allowing efficiency to modulate over trials (i.e. frequency-fixed). Δfreq = regression model of predicted scores computed allowing frequency to modulate over trials (i.e. efficiency-fixed). **p<.01**,** ***p<.001.

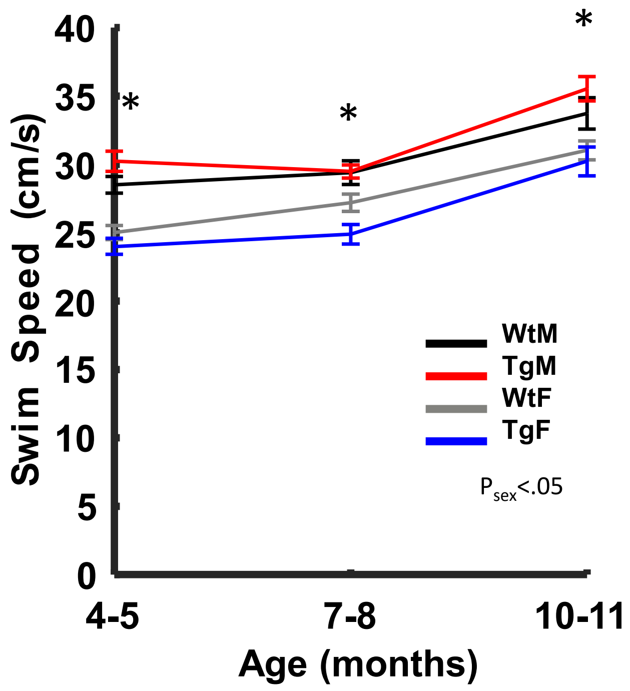

Figure S5. Average swim speed (cm/s) is plotted for each age of testing. Note that males consistently swam faster than females across all age points (ps<.05, ANOVAs). Groups distinguished by color: black=Wild Type males (WtM), red=Transgenic males (TgM), gray=Wild Type females (WtF), blue=Transgenic females (TgF). Data shown in mean ± SEM.

	4-5mo		7-8mo		10-11mo	
	Mean	*SEM*	*Mean*	*SEM*	*Mean*	*SEM*
WT Males	.030	±.002	.050	±.003	.070	±.001
Tg+/- Males	.081	±.002	.043	±.001	.081	±.002
WT Females	.100	±.001	.083	±.004	.042	±.002
Tg+/- Females	.044	±.002	.025	±.001	.070	±.002

Figure S6. Table showing the means and SEM on non-spatial errors at each age of testing

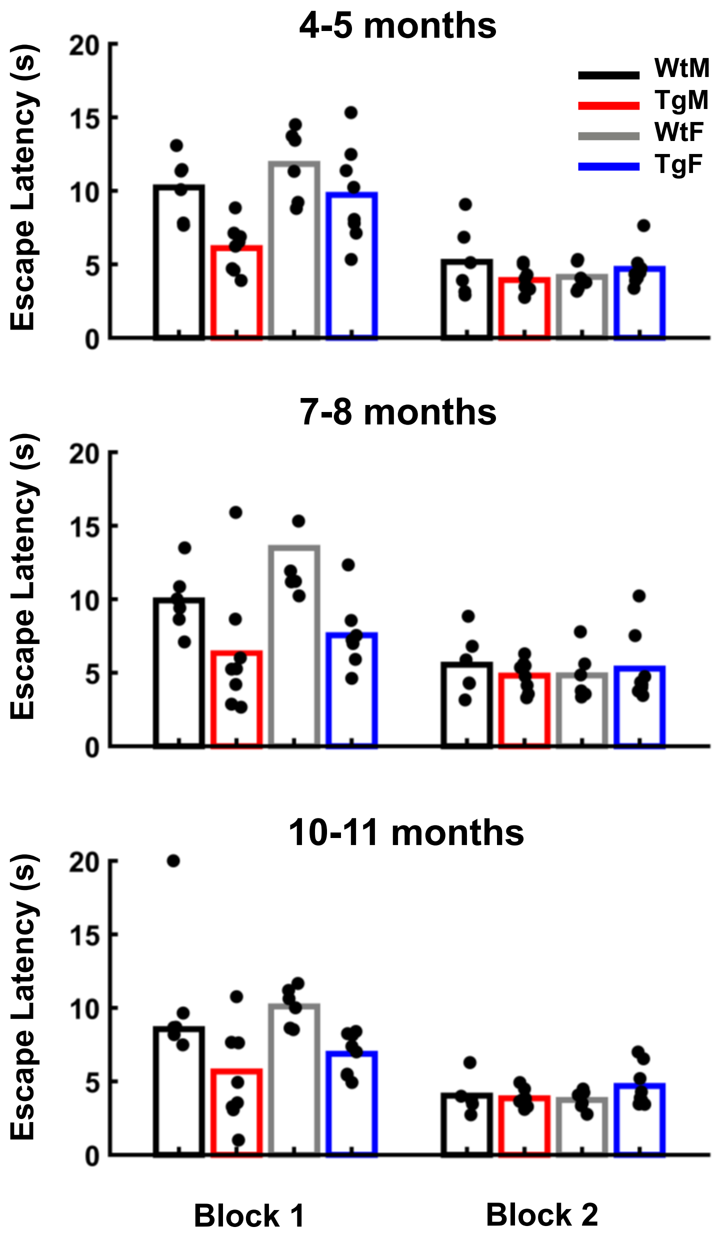

Figure S7. Mean escape latency (s) across trial blocks (set of 4 trials per block) during cued task was not different between groups at each age of testing. Mean latencies are plotted for each animal (black circles) to show distribution. Groups distinguished by color: black=Wild Type males (WtM), red=Transgenic males (TgM), gray=Wild Type females (WtF), blue=Transgenic females (TgF). Bars represent mean values. Black dots indicate raw data. Escape latency data for the 4-5 month time point have been published previously in Pentkowski et al., 2017.
